# Comparison between Different Bulk-Fill and Incremental Composite Materials Used for Class II Restorations in Primary and Permanent Teeth: In Vitro Assessments

**DOI:** 10.3390/ma16206674

**Published:** 2023-10-13

**Authors:** Maria Salem Ibrahim, Ahmed Saleh AlKhalefah, Abdullah Ali Alsaghirat, Read Ahmed Alburayh, Nezar Ahmed Alabdullah

**Affiliations:** 1 Department of Preventive Dental Sciences, College of Dentistry, Imam Abdulrahman Bin Faisal University, Dammam 34212, Saudi Arabia; 2 College of Dentistry, Imam Abdulrahman Bin Faisal University, Dammam 31441, Saudi Arabia; alkhalefahahmed25@gmail.com (A.S.A.); saghirat98@gmail.com (A.A.A.); 2170002781@iau.edu.sa (R.A.A.); dr.nezaralabdullah@gmail.com (N.A.A.)

**Keywords:** Bulk Fill, incremental, composite, microleakage, class II restoration, bioactive, packable composite

## Abstract

Introduction: Several advantages, including improved aesthetics and conservative cavity preparation, made resin-based composite (RBC) a popular restorative material. However, several limitations come with RBC restorations such as the necessity for proper isolation of the tooth and an incremental layering for the material due to the limitations of the depth of cure. Despite these advantages and limitations, the usage of these restorative materials is increasingly being expanded due to the advancement made since their introduction. To overcome some of the limitations, several types of RBC restorations were developed. Materials and Methods: Four different RBC materials used for class II restorations in primary and permanent teeth were compared: Z350 XT Filtek™ Universal Restorative (ZXT), Filtek™ Bulk Fill Flowable Restorative (FBF), Beautifil-Bulk Flowable (BBF) and Tetric™ N-Flow (TNF). Flexure strength, elastic modulus, surface roughness, microhardness and microleakage were assessed using standard methods or previously published protocols. The data and differences between the groups were analyzed using One-way analysis of variance (ANOVA), Tukey’s multiple comparisons, Kruskal–Wallis and Wilcoxon rank-sum (Mann–Whitney) tests. Results: The study found that BBF (86.24 ± 7.41 MPa) and ZXT (64.45 ± 11.52 MPa) had higher flexural strength than FBF (50.89 ± 8.44 MPa) and TNF (50.67 ± 9.40 MPa), while both exhibited the highest values of surface roughness. Elastic modulus was the highest with BBF, which was not statistically significant from FBF or ZXT (*p* > 0.05). ZXT (109.7 ± 7.83 VH) exhibited the highest value of microhardness, which was statistically significant from the other three materials (*p* < 0.0001). Microleakage was assessed after thermocycling for 20,000 cycles to simulate two years in the mouth. FBF (70%) exhibited the most resistance to microleakage. Conclusions: Different types of RBC restorations exhibit different characteristics. The clinician needs to choose the most appropriate restorative material based on different clinical scenarios.

## 1. Introduction

The usage of photopolymerizable resin-based composite restorative materials (RBCs) is increasingly being expanded to large and deep cavities due to a significant advancement since their introduction in the 1960s [1]. However, its effectiveness may vary depending on the depth of cure and its suitability to potentially reduce the consequences of shrinkage stresses. Incremental techniques are commonly used to overcome polymerization shrinkage [2,3,4,5,6]. This technique, however, is time-consuming and may result in air entrapment between successive layers of composite resin [2,7,8]. Some chemical and structural changes in the composite resin composition have been proposed to reduce the undesirable effects of RBCs. Modifications to the resin matrix, inorganic particle quantity, shape or surface treatment are examples of modifications to reduce the undesirable effects [9,10,11].

RBCs have several advantages over dental amalgam, including improved aesthetics. RBCs are adhesively bonded to the tooth using a compatible bonding system, allowing for conservative cavity preparation. RBCs longevity as a material for restoring both Class I and II cavities is being supported by many studies [8,9]. The use of posterior RBC restorations can have some drawbacks. Proper isolation of the tooth is required, and an incremental layering technique is currently recommended [8,9].

The composite material market is frequently driven by consumers’ desire for faster and easier materials by reducing curing time and/or using larger composite layers [2]. Furthermore, dental practitioners are becoming more interested in time-saving Bulk-Fill RBC restorations, which can be placed in increments of 4 mm and cured in one cycle of light curing. This will skip the time-consuming layering process and have a proper viscosity for easier application in the tooth cavity [8,12,13].

Low-viscosity Bulk-Fill resin composites were the first materials developed. These flowable materials are indicated as a restorative base that requires a 2 mm thick layer of the conventional resin composite [14,15,16]. Later, “Full-Body” Bulk-Fill restorative resin composites, which resemble paste, were introduced. These materials can be used in high-masticatory load-bearing areas without the need for coverage because they have a higher percentage of the inorganic fillers [14,15,16].

The main component of Bulk-Fill resin composites is conventional methacrylate monomer pre-polymerized particles [15]. Modified monomers can be used to produce materials with low polymerization shrinkage stresses and improved physical and mechanical properties. These particles, which are included in Bulk-Fill resin as aromatic urethane dimethacrylate (AUDMA) and addition fragmentation monomers (AFM), act as chemical modulators of the polymerization reaction [15].

Incomplete polymerization is one of the most prevalent problems with flowable Bulk-Fill composites, as it is with all RBCs [17]. In clinical use, the polymerized resin composite may contain a sizable amount of leftover dimethacrylate monomers [17]. Such monomers can diffuse into the pulp through dentinal tubules or elute from polymerized dental methacrylate-based materials into the oral cavity [17]. The structural stability and biocompatibility of the substance may be impacted by the leaching of monomers [17].

The primary improvement of Bulk-Fill materials is an elevated depth of cure, to allow for greater light penetration [18]. Some manufacturers have reduced the number of glass fillers used. Others, on the other hand, have introduced large filler particles with a low surface area to reduce light reflection and scattering. Low shrinkage stress is associated with changes in the filler content and/or the organic matrix. Ideally, these perceived improvements should not degrade the material’s mechanical properties. According to recent studies, Bulk-Fill resin composites exhibit acceptable levels of creep resistance in the range of conventional material types [2,19,20,21].

With the advancements in Bulk-Fill and incremental composite materials, dentists have many options to restore primary and permanent teeth. Depending on the needed properties, dentists can judge each material to choose the most efficient and effective material for each case. Although many studies have assessed the material properties of Z350 XT Filtek™ Universal Restorative (ZXT) composite, there is a lack of studies comparing this material with newer materials in the market such as Filtek™ Bulk Fill Flowable Restorative (FBF) and Beautifil-Bulk Flowable (BBF) composites. This gap may limit our understanding of the properties of these materials. The objectives of this study were to measure and compare some of the properties of ZXT, FBF, BBF and Tetric™ N-Flow (TNF).

## 2. Materials and Methods

### 2.1. Ethical Approval and Study Design

**Figure 1:**
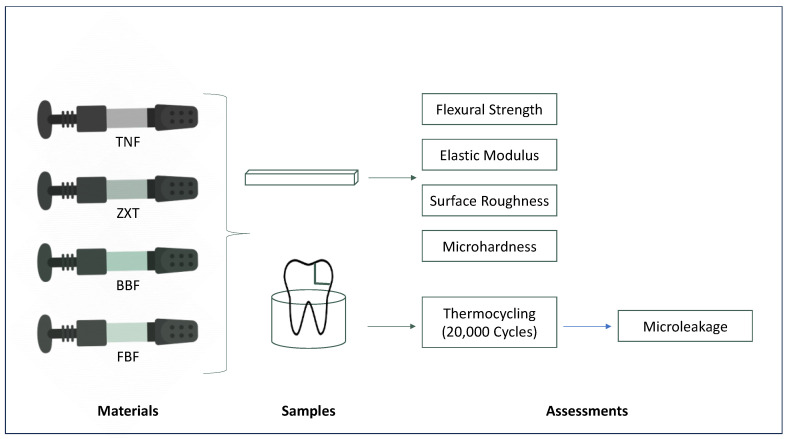
Flow-chart summarizing the study materials and methods.

This study is an in vitro study that was conducted at the College of Dentistry Research Laboratory at Imam Abdulrahman bin Faisal University. Approval was obtained from the Institutional Review Board (IRB-2021-02-484) prior to start. The study employed recently extracted teeth. The restorative materials compared in this study are listed in Table 1. A flow-chart that summarizes the study is presented in Figure 1.

### 2.2. Flexural Strength and Elastic Modulus

#### 2.2.1. Sample Preparation

Samples (*n* = 10) were prepared using a 2 × 2 × 25 mm stainless steel mold following the ISO 4049 [22]. Before placement of the material, the mold was covered with Mylar strips and glass slides from below, and the material was injected in the mold; following that, another Mylar strip and glass slide were placed at the top, and the slides were pressed to eliminate any air bubbles. The curing for all samples was conducted using a light-emitting source (Satelec Mini LED Curing Light 1250 mW/cm^2^, A-dec Inc., Newberg, OR, USA) for 20 s [12,23,24].

#### 2.2.2. Test

**Figure 2:**
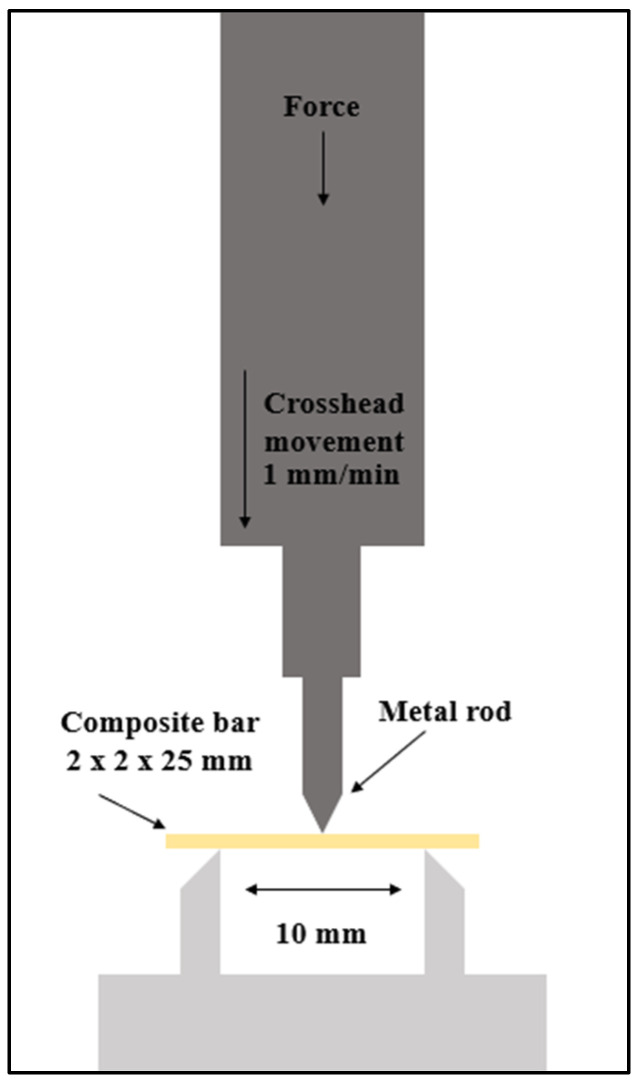
Flexural strength and elastic modulus testing illustration.

The flexural strength and elastic modulus were measured via three-point flexure with a 10 mm span using the universal testing machine (NSTRON 5965 load frame, Boston, MA, USA) at a crosshead speed of 1 mm/min shown in (Figure 2) [23,24].

Flexural strength was calculated using the following the formula [23,24]:

(1) $$F=\frac{3LS}{2WH^{2}}$$

whereby F = flexural strength, L = maximum load, S = span, W = width of the specimen and H = height.

Elastic modulus was calculated using the following the formula:

(2) $$M=\frac{LS^{3}3}{4WH^{3}3d}$$

whereby M = elastic modulus, L = maximum load, S = span, W = width of the specimen, H = height and d = defluxion corresponding to the load (L).

### 2.3. Microhardness

#### 2.3.1. Sample Preparation

Samples (*n* = 10) were prepared in the same method mentioned in Section 2.2.1.

#### 2.3.2. Test

A Microhardness tester (BUEHLER MicroMet 6040 Hardness Tester, Shanghai, China) was used to obtain the surface microhardness of the tested materials. The surface microhardness was calculated by averaging three 100 μm spaced indentations using a Vickers indenter head with 25 g force for 10 s on the samples, then the size of each indentation was measured using a built-in microscope [25,26].

### 2.4. Surface Roughness

#### 2.4.1. Sample Preparation

Samples (*n* = 10) were prepared in the same method mentioned in Section 2.2.1.

#### 2.4.2. Test

Surface roughness was evaluated using a three-dimensional high-resolution non-contact optical profiler (Contour GT-K 3D Optical Microscope, Bruker, Billerica, MA, USA). The samples were scanned at 20× magnification, at a scan speed of 1× and threshold of 5% [26]. The software Vision 64 (Bruker, Billerica, MA, USA) was used for the analysis and graphical output, and the average of three scans for every sample was taken [25]. The average surface roughness (Ra) was reported as the arithmetical mean of roughness profile [26].

### 2.5. Microleakage

#### 2.5.1. Sample Preparation

The extracted teeth were inspected to ensure that the part that would be restored is non-carious. After inspection, teeth were stored in 0.01% (*w*/*v*) thymol solution (pH 7.0) until use. Teeth were mounted vertically using self-curing acrylic resin (Unifast III, GC Corporation, Tokyo, Japan), exposing the crowns until 2 mm below the cementoenamel junction. After mounting, a box preparation (Class II) was conducted on the proximal surfaces of the teeth with the following dimensions: 4 mm occlusogingival, 2 mm mesiodistal and 2 mm buccolingual. One standardized operator prepared all teeth, then the prepared teeth were randomly assigned to the study’s groups. Before the restorations were placed, etching was conducted using 35% phosphoric acid (Cica Etching Gel, Neumünster, Germany) for 15 s and then dried for 5 s, and bonding was applied using self-priming resin (3M ESPE Single Bond Universal Adhesive, Neuss, Germany) and was cured for 20 s. Following etching and bonding, the restorations were placed using a single operator, and the curing was conducted using a light-emitting source (Satelec Mini LED Curing Light 1250 mW/cm^2^, A-dec Inc., Newberg, OR, USA) for 20 s.

#### 2.5.2. Test

The prepared samples (*n* = 8–10) of all groups were put in thermocycling between 5° and 55° for 20,000 cycles to simulate two years in the mouth [27]. After thermocycling was completed, nail varnish was used to paint the tooth with two coats within 1 mm of the restoration margin, then the tooth was immersed in methylene blue 1% for 2 h [13,28,29]. After 24 h, the samples were washed for 5 min with abundant water and then were sectioned mesiodistally and were checked under a stereomicroscope. Teeth were scored based on the degree of dye penetration (Table 2) [29]. Scoring was conducted by three trained examiners for all samples. If conflict was noticed in any score, the sample was re-evaluated and re-scored by agreement.

### 2.6. Statistical Analysis

The Shapiro–Wilk test was used to check the data normality. One-way analysis of variance (ANOVA) was used to analyze the results of flexural strength, elastic modulus, microhardness and surface roughness. Multiple comparisons between the studied groups were conducted using Tukey’s multiple comparison test. The Kruskal–Wallis test and Wilcoxon rank-sum (Mann–Whitney) test were used to analyze the microleakage scores. All the statistical analyses were performed with GraphPad Prism Version 9.5.1 (GraphPad Software, Boston, MA, USA) and Stata/IC 14.2 (Stata, College Station, TX, USA).

## 3. Results

### 3.1. Flexural Strength and Elastic Modulus

**Figure 3:**
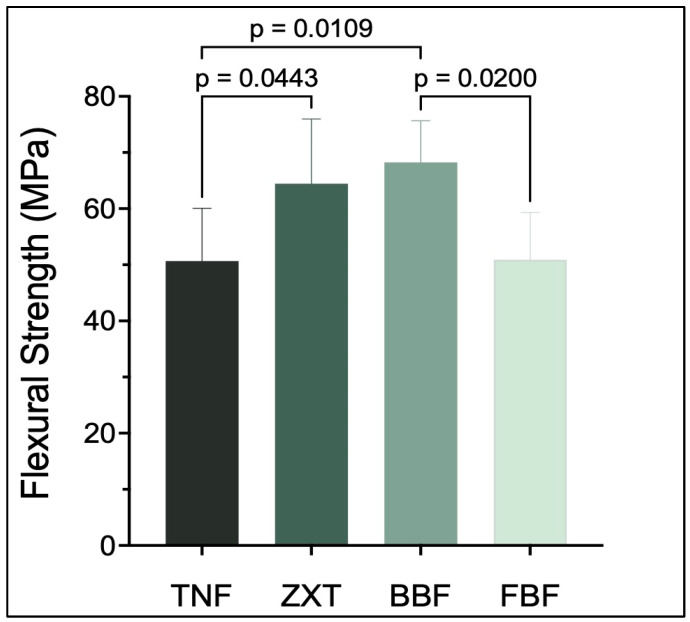
Flexural strength (mean ± SD) of the studied groups. *p*-value is denoted on the graph only if there is a significant difference between the two groups (*p* < 0.05).

Beautifil-Bulk Flowable has the highest flexural strength, while Tetric N-Flow has the lowest. There are statistically significant differences (*p* < 0.05) between Tetric N-Flow and Z350 XT Filtek Universal Restorative, between Tetric N-Flow and Beautifil-Bulk Flowable and between Beautifil-Bulk Flowable and Filtek Bulk Fill Flowable Restorative as shown in Table 3 and Figure 3.

**Figure 4:**
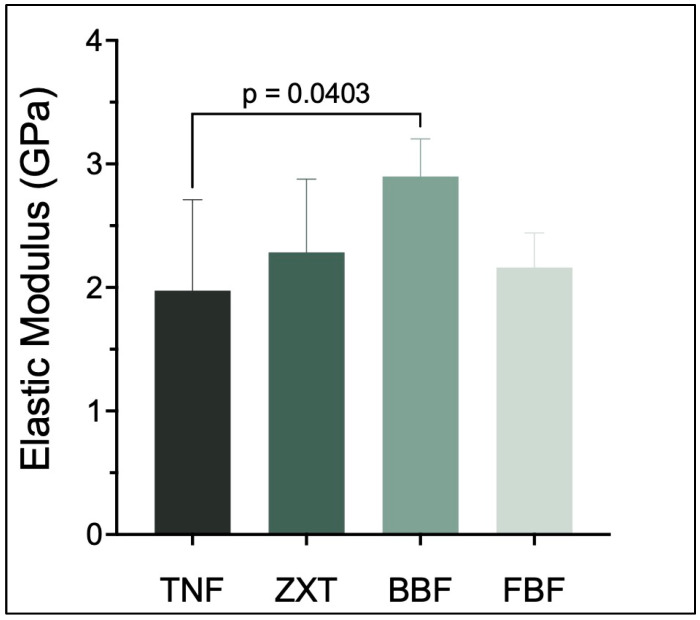
Elastic modulus (mean ± SD) of the studied groups. *p*-value is denoted on the graph only if there is a significant difference between the two groups (*p* < 0.05).

Tetric N-Flow has the lowest elastic modulus in comparison to other tested materials. Its elastic modulus was statistically and significantly different than Beautifil-Bulk Flowable (*p* < 0.05) (Table 4 and Figure 4).

### 3.2. Surface Roughness

**Figure 5:**
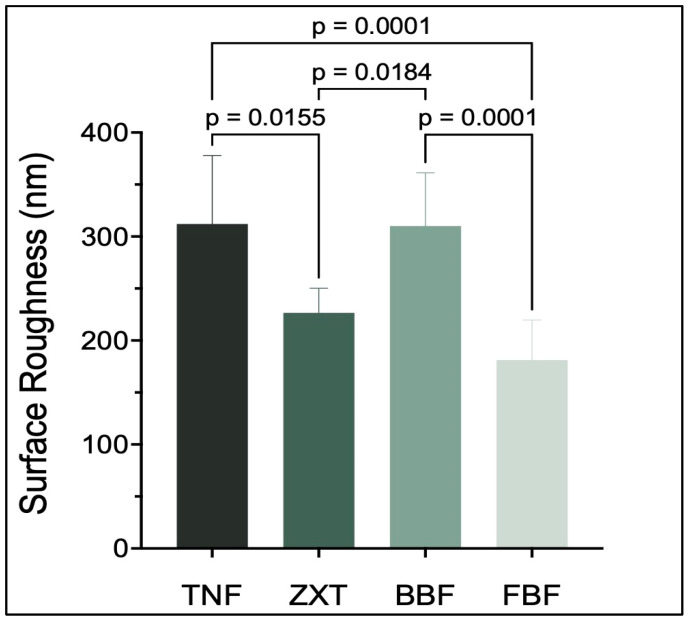
Surface roughness (Ra) (mean ± SD) of the studied groups. *p*-value is denoted on the graph only if there is a significant difference between the two groups (*p* < 0.05).

Tetric N-Flow and Beautifil-Bulk Flowable have the highest values of surface roughness, while Filtek Bulk Fill Flowable Restorative has the lowest value. There are statistically significant differences (*p* < 0.05) between Tetric N-Flow and Filtek Bulk Fill Flowable Restorative, between Beautifil-Bulk Flowable and Z350 XT Filtek Universal Restorative, between Tetric N-Flow and Z350 XT Filtek Universal Restorative and between Beautifil-Bulk Flowable and Filtek Bulk Fill Flowable Restorative as shown in Table 5 and Figure 5.

### 3.3. Microhardness

**Figure 6:**
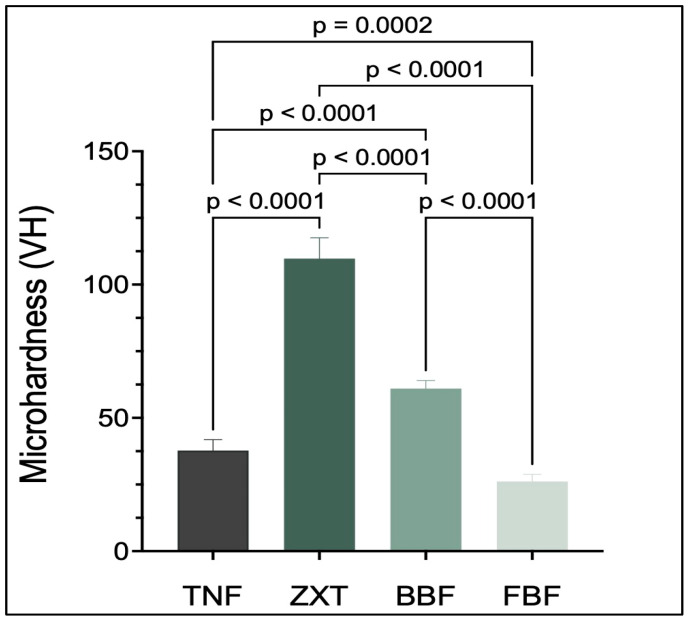
Microhardness values (mean ± SD) of the studied groups. *p*-value is denoted on the graph only if there is a significant difference between the two groups (*p* < 0.05).

Z350 XT Filtek Universal Restorative has the highest microhardness, while Filtek Bulk Fill Flowable Restorative has the lowest. There are statistically significant differences (*p* < 0.05) between Tetric N-Flow and Filtek Bulk Fill Flowable Restorative, between Z350 XT Filtek Universal Restorative and Filtek Bulk Fill Flowable Restorative, between Tetric N-Flow and Beautifil-Bulk Flowable, between Z350 XT Filtek Universal Restorative and Beautifil-Bulk Flowable, between Tetric N-Flow and Z350 XT Filtek Universal Restorative and between Beautifil-Bulk Flowable and Filtek Bulk Fill Flowable Restorative as shown in Table 6 and Figure 6.

### 3.4. Microleakage

Most (80–88%) of the samples of the three groups—Tetric N-Flow, Z350 XT Filtek Universal Restorative and Beautifil-Bulk Flowable—scored 3 in microleakage dye penetration. In contrast, Filtek Bulk Fill Flowable Restorative samples, mostly (70.0%), scored 0 in microleakage dye penetration as shown in Table 7. The Kruskal–Wallis rank test showed that there was a statistically significant difference in microleakage score between the different groups (chi-squared with ties = 11.806 and *p* = 0.0081). The Wilcoxon rank-sum (Mann–Whitney) test showed statistically significant differences between Filtek Bulk Fill Flowable Restorative and the other three groups—Tetric N-Flow, Z350 XT Filtek Universal Restorative and Beautifil-Bulk Flowable (*p* = 0.0074, *p* = 0.0066 and *p* = 0.0285, respectively).

## 4. Discussion

Proximal cavities and class II restorations in primary and permanent teeth are sensitive procedures that need careful preparation and selection of the restorative material. Proper selection of the materials to restore these cavities depends on various factors such as cavity depth, shape and area and opposing tooth condition [30]. Also, patient-related factors such as cooperation, caries risk and financial status are other factors that should be considered while selecting the restorative materials [30]. In this study, some recently introduced materials in the dental market are compared in terms of flexural strength, elastic modulus, surface roughness, surface microhardness and microleakage. These materials included the Z350 XT Filtek™ Universal Restorative, which is considered as a universal packable composite; Filtek™ Bulk Fill Flowable Restorative, which is considered as a Bulk-Fill flowable composite; Beautifil-Bulk Flowable, which is considered as a bioactive Bulk-Fill flowable composite as well as Tetric™ N-Flow, which is considered as a conventional flowable composite.

The degradation of mechanical properties in the oral environment, combined with the growth and accumulation of cracks, leads to the catastrophic failure of restorations [31]. RBCs must be high in strength to withstand repeated chewing forces. In vitro, flexural strength testing has been demonstrated to be an effective method for determining the strength of a restorative material [30]. Previous studies suggested that Bulk-Fill RBCs generally have a higher flexural strength than their flowable counterparts [15,32]. This was reflected in this study as the Bulk-Fill materials tested demonstrated a higher flexural strength than their conventional flowable composite. Beautifil-Bulk Flowable showed the highest flexural strength, followed by Z350 XT Filtek Universal Restorative (Conventional packable Class I-6), followed by Filtek Bulk Fill Flowable Restorative and Tetric N-Flow.

The elastic modulus, also known as Young’s modulus, is a value used to measure a material’s rigidity and is defined as the stress/strain ratio in an elastic state [33]. The elastic modulus is affected by the filler content. Higher filler contents lead to a higher elastic modulus, which means the material will have a greater ability to resist deformation [34]. It should be noted that a low elastic modulus material should not be used in stress-bearing areas as their abilities to resist deformities are lower. The Tetric N-Flow, being a conventional flowable composite, demonstrated the lowest elastic modulus.

A dental restorative material’s surface structure and composition influence initial bacterial adhesion, and a rough material surface will accumulate more plaque [35]. The surface roughness of a material is influenced by its composition, particles or filler type, shape, size and amount [35]. In this study, Tetric N-Flow and Beautifil-Bulk Flowable showed the highest values of surface roughness, followed by Z350 XT Filtek Universal Restorative, while Filtek Bulk Fill Flowable Restorative had the lowest surface roughness. The increased surface roughness of Beautifil-Bulk Flowable could be due to its composition and the surface’s pre-reacted glass-ionomer particles [26]. However, the bioactivity [26] of these particles can be considered as an advantage that can overcome the increase in surface roughness.

The surface hardness of enamel refers to dental resistance to scratches, abrasion and indentation, as well as resistance to permanent curvature and deformation during force application [36]. Microhardness can be increased by increasing the curing time and intensity of the curing light [37]. Previous studies showed different curing depths for Bulk-Fill composites depending on the techniques used or the type [2]. In a previous study, Bulk-Fill and conventional packable composites showed higher microhardness than flowable composites [38]. In this study, where the curing time and intensity were controlled and fixed for all tested materials, Z350 XT Filtek Universal Restorative showed the highest microhardness, followed by Beautifil-Bulk Flowable then Tetric N-Flow, while Filtek Bulk Fill Flowable Restorative showed the lowest microhardness. There were statistically significant differences between the materials in which they were arranged from highest to lowest.

Microleakage in dental restorations is the formation of gaps at the tooth-restoration interface [39]. Although, restorations are placed to protect the remaining tooth structure and to seal and protect the exposed dentin or pulp; some restorations will deteriorate over time and result in gap formations [40]. In this study, Tetric N-Flow, Z350 XT Filtek Universal Restorative and Beautifil-Bulk Flowable showed high scores of microleakage after 20,000 cycles of thermocycling, which simulates two years in the mouth. Although the bioactive flowable Bulk-fill showed a high score for microleakage, it was previously reported that Activa kids, which is a bioactive flowable composite, had low microleakage [41]. However, in that study, the samples were only exposed to 200 cycles of thermocycling [41]. In another study, an Activa bioactive restorative showed a comparable result in microleakage to conventional composites [42]. Also, the microleakage in the bioactive flowable Bulk-Fill after thermocycling could be explained by the release of the ions from the material over time, leaving gaps in the restoration that lead to microleakage. Previous studies suggested that with the increasing viscosity of the materials, there is an increase in marginal gap formation [2]. This could explain the resistance to microleakage reported in this study from the flowable Bulk-Fill material in comparison to the packable material.

Further studies are necessary to assess other properties of these materials, such as the tensile strength, fracture pattern, toxicity, wear resistance, color stability, degree of conversion and depth of cure.

## 5. Conclusions

Different types of RBC restorations exhibit different physical and mechanical characteristics. In this study, BBF and ZXT showed the highest flexural strength and surface roughness. BBF showed the highest elastic modulus but was not significantly different from the ZXT. ZXT showed the highest microhardness, while FBF showed the highest resistance to microleakage after 20,000 cycles of thermocycling, which resembles 2 years in the mouth. A clinician must choose the appropriate materials based on the clinical findings and needs. With the advancement in material development, further advancements in composite systems are expected. However, careful judgment of the properties of each material is important.

## Figures and Tables

**Table 1:** Materials used in the study.

Material	Composition	Manufacturer	Country of Origin	Abbreviation
Z350 XT Filtek™ Universal Restorative	Bis-GMA, bis-EMA, UDMA, TEGDMA, silica filler, zirconia filler, zirconia/silica cluster filler	3M	Saint Paul, MN, USA	ZXT
Filtek™ Bulk Fill Flowable Restorative	Bis-GMA, bis-EMA, UDMA, procrylat resins, zirconia/silica filler, ytterbium trifluoride filler	3M	Saint Paul, MN, USA	FBF
Beautifil-Bulk Flowable	Bis-GMA, bis-MPEPP, UDMA TEGDMA, S-PRG fillers	Shofu INC.	Kyoto, Japan	BBF
Tetric™ N-Flow	Bis-GMA, UDMA, TEGDMA, silicon dioxide, mixed oxide, ytterbium trifluoride filler, barium glass	Ivoclar Vivadent	Schaan, Liechtenstein	TNF

Bis-GMA: Bisphenol-A glycidyl dimethacrylate, Bis-EMA: Bisphenol-A ethoxylated dimethacrylate, UDMA: Urethane dimethacrylate, TEGDMA: Triethylene glycol dimethacrylate, Bis-MPEPP: Bisphenol A polyethoxy methacrylate and S-PRG: Surface Pre-reacted Glass-ionomer.

**Table 2:** Scoring system used for assessing microleakage with dye penetration.

Score	Degree of Dye Penetration
0	No dye penetration
1	Dye penetration less than half of the axial/gingival wall
2	Dye penetration more than half of the axial/gingival wall
3	Dye penetration spreading along the axial/gingival wall

**Table 3:** Mean differences and confidence interval differences for flexural strength.

Tukey’s Multiple Comparisons Test	Mean Difference	95.00% CI of Difference	Below Threshold?	Summary	Adjusted *p* Value
TNF vs. ZXT	−13.79	−27.29 to −0.2774	Yes	*	0.0443
TNF vs. BBF	−17.57	−31.67 to −3.475	Yes	*	0.0109
TNF vs. FBF	−0.2271	−14.32 to 13.87	No	ns	>0.9999
ZXT vs. BBF	−3.785	−18.31 to 10.74	No	ns	0.8876
ZXT vs. FBF	13.56	−0.9624 to 28.08	No	ns	0.0731
BBF vs. FBF	17.34	2.275 to 32.41	Yes	*	0.0200

CI: confidence interval, ns: not significant, *: *p* < 0.05.

**Table 4:** Mean differences and confidence interval differences for elastic modulus.

Tukey’s Multiple Comparisons Test	Mean Difference	95.00% CI of Difference	Below Threshold?	Summary	Adjusted *p* Value
TNF vs. ZXT	−0.3095	−1.153 to 0.5339	No	ns	0.7358
TNF vs. BBF	−0.9234	−1.814 to −0.03318	Yes	*	0.0403
TNF vs. FBF	−0.1867	−1.077 to 0.7036	No	ns	0.9349
ZXT vs. BBF	−0.6139	−1.560 to 0.3317	No	ns	0.2947
ZXT vs. FBF	0.1228	−0.8228 to 1.068	No	ns	0.9831
BBF vs. FBF	0.7368	−0.2509 to 1.724	No	ns	0.1910

CI: confidence interval, ns: not significant, *: *p* < 0.05.

**Table 5:** Mean differences and confidence interval differences for surface roughness.

Tukey’s Multiple Comparisons Test	Mean Difference	95.00% CI of Difference	Below Threshold?	Summary	Adjusted *p* Value
TNF vs. ZXT	85.47	13.45 to 157.5	Yes	*	0.0155
TNF vs. BBF	1.953	−62.46 to 66.37	No	ns	0.9998
TNF vs. FBF	130.8	61.98 to 199.7	Yes	***	0.0001
ZXT vs. BBF	−83.52	−155.5 to −11.49	Yes	*	0.0184
ZXT vs. FBF	45.37	−30.65 to 121.4	No	ns	0.3777
BBF vs. FBF	128.9	60.02 to 197.8	Yes	***	0.0001

CI: confidence interval, ns: not significant, ***: *p* < 0.001, *: *p* < 0.05.

**Table 6:** Mean differences and confidence interval differences for microhardness.

Tukey’s Multiple Comparisons Test	Mean Difference	95.00% CI of Difference	Below Threshold?	Summary	Adjusted *p* Value
TNF vs. ZXT	−72.02	−78.42 to −65.61	Yes	****	<0.0001
TNF vs. BBF	−23.26	−29.67 to −16.86	Yes	****	<0.0001
TNF vs. FBF	11.58	5.176 to 17.99	Yes	***	0.0002
ZXT vs. BBF	48.76	42.17 to 55.35	Yes	****	<0.0001
ZXT vs. FBF	83.60	77.01 to 90.19	Yes	****	<0.0001
BBF vs. FBF	34.84	28.25 to 41.43	Yes	****	<0.0001

CI: confidence interval, ns: not significant, ****: *p* < 0.0001, ***: *p* < 0.001.

**Table 7:** Microleakage test scoring results.

Groups	Score 0 *n* (%)	Score 1 *n* (%)	Score 2 *n* (%)	Score 3 *n* (%)	Total *n* (%)
TNF	0 (0.0)	0 (0.0)	1 (12.5)	7 (87.5)	8 (100.0)
ZXT	0 (0.0)	2 (20.0)	0 (0.0)	8 (80.0)	10 (100.0)
BBF	2 (20.0)	0 (0.0)	0 (0.0)	8 (80.0)	10 (100.0)
FBF	7 (70.0)	0 (0.0)	0 (0.0)	3 (30.0)	10 (100.0)

## Data Availability

The data presented in this study are available on request from the corresponding author.
